# Supplementary material for: Multi-resolution deep learning characterizes tertiary lymphoid structures and their prognostic relevance in solid tumors
Source: Commun Med (Lond). 2024 Jan 5;4:5. doi: 10.1038/s43856-023-00421-7 (PMC10770129; doi:10.1038/s43856-023-00421-7)
Supplement: Supplementary file 2 — Supplementary Information [file 43856_2023_421_MOESM2_ESM.pdf]

# Supplementary Figures

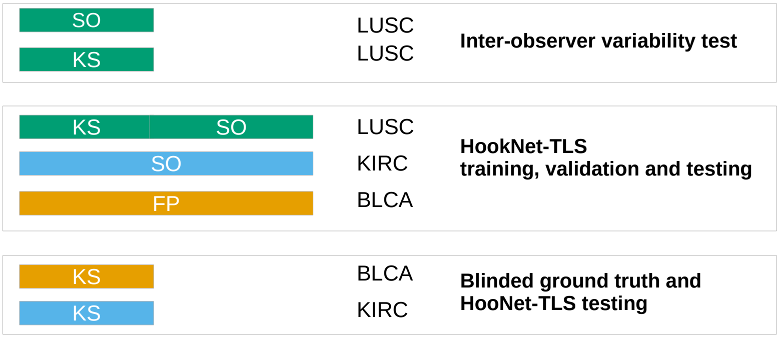


**Supplementary Figure 1** Schematic representation of image annotation datasets created by three trained researchers. All the Cancer Genome Atlas (TCGA) lung squamous cell carcinoma (LUSC) slides were annotated by SO. A random selection of 133 LUSC slides was also annotated by KS: 44 slides were part of the test set, 24 slides were used as the validation set and 65 were part of the training set. The 44 TCGA LUSC test set slides annotated by KS and SO were used for the inter-observer variability assessment as a comparison for HookNet-TLS performance. The TCGA clear cell renal cancer (KIRC) cohort was annotated solely by SO and the TCGA muscle invasive bladder cancer (BLCA) cohort - solely by FP. The independent LUSC and KICR slide sets from University Hospital Zurich (USZ) were annotated by KS. Different annotators were assigned for different tumor types to mimic the real-life situation of tertiary lymphoid structure (TLS) assessment in different clinical centers.

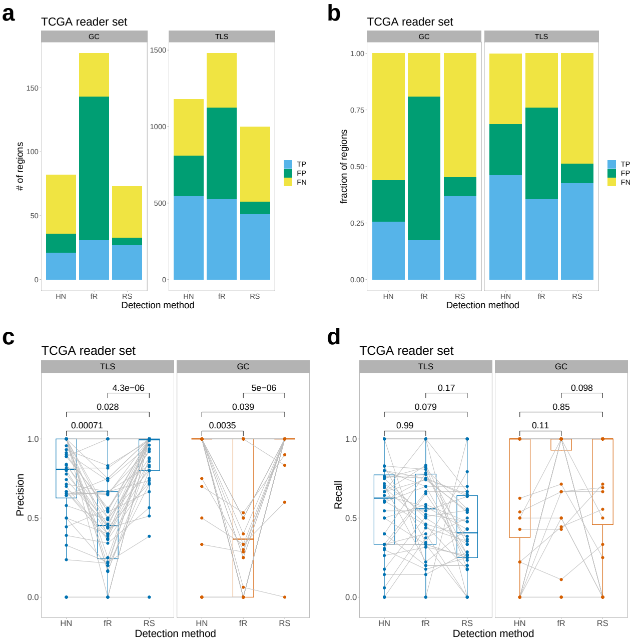


**Supplementary Figure 2** Comparison of deep learning models and second reader in reader study (RS) set (n=44). a: Number of tertiary lymphoid structure (TLS) and germinal center (GC) regions predicted as true positive (TP), false negative (FN) or false positive (FP) in the Cancer Genome Atlas (TCGA) reader set images. b: Fraction of TLS and GC regions predicted as true positive (TP), false negative (FN) or false positive (FP) in the TCGA reader set images. c: Precision metric was measured in slide of the TCGA reader study and compared between HookNet, Faster-RCNN and inter-observer variability by paired Mann Whitney U test. d: Recall metric was measured in slide of the TCGA reader study and compared between HookNet, Faster-RCNN and inter-observer variability by paired Mann Whitney U test. Boxes in c and d span across the 25th and 75th percentiles, and whiskers (Tukey style) extend from the hinge to the largest value no further than 1.5 * interquartile range.

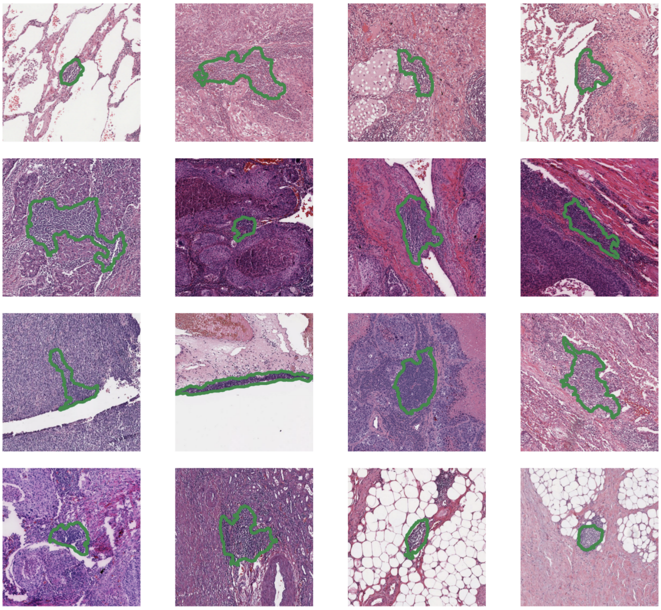

**Supplementary Figure 3** A representative set of detections from HookNet-TLS, which appear to be true tertiary lymphoid structures (TLSs) based on visual inspection but that were not included in the manual annotations.

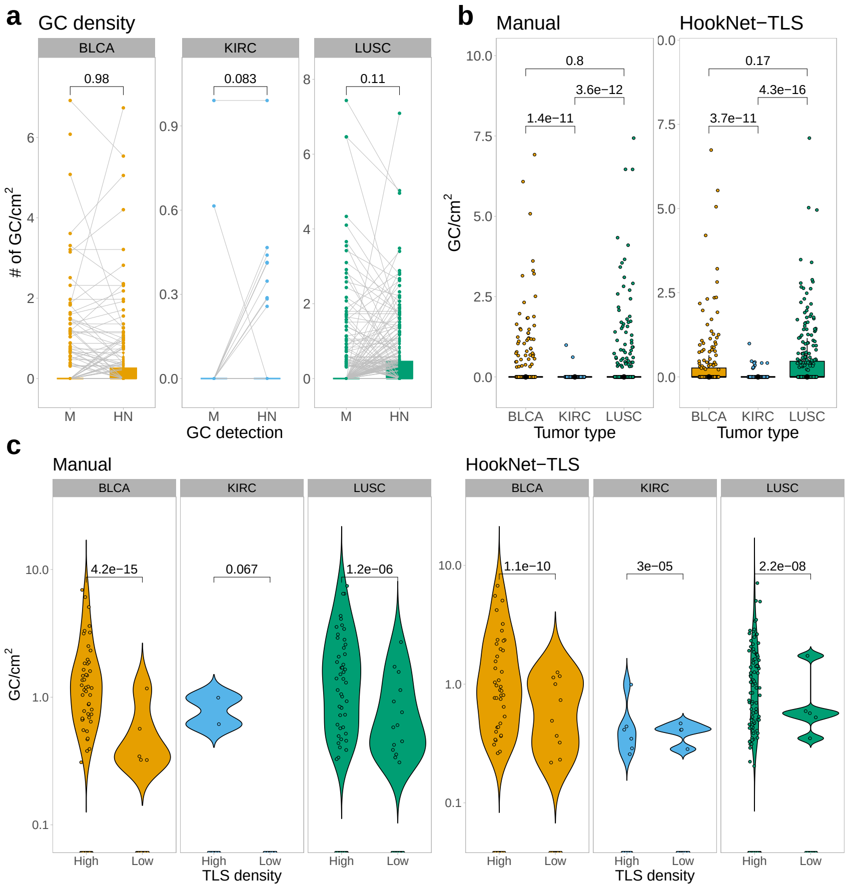


**Supplementary Figure 4** Analysis of GC parameters in different tumors. GC parameters were assessed in the Cancer Genome Atlas (TCGA) test set slides (n=767). a: Pairwise comparison of manually annotated versus HookNet-TLS predicted germinal center (GC) density in each image by paired Mann-Whitney U test. b: GC density compared across organs by Mann-Whitney U test obtained by manual or HookNet-TLS detection. Boxes in a and b span across the 25th and 75th percentiles, and whiskers (Tukey style) extend from the hinge to the largest value no further than 1.5 * interquartile range. c: Median tertiary lymphoid structure (TLS) density in each tumor type was used as a cutoff to define TLS-high and TLS-low tumors. GC density was compared between the TLS density groups by Mann-Whitney U test.

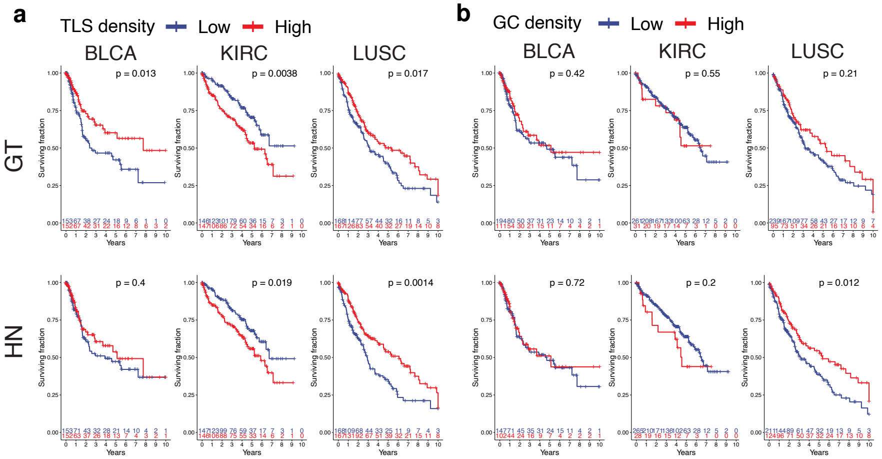


**Supplementary Figure 5** Analysis of the prognostic relevance of tertiary lymphoid structures (TLSs) and germinal centers (GCs) in whole cohorts (train+validation+test, n=1019). Overall survival was compared between patient groups by Kaplan Meier curves and log-rank test. A: Groups were defined by median TLS densities of whole tumor cohorts of each tumor type for manually annotated (GT) and predicted TLS (HN). B: Groups were defined by median GC densities of whole cohorts of each tumor type for manually annotated (GT) and predicted TLS (HN). Due to the lower frequency of GCs, this threshold corresponded to GC-negative vs GC-positive tumors. GT, ground truth; HN, HookNet-TLS.

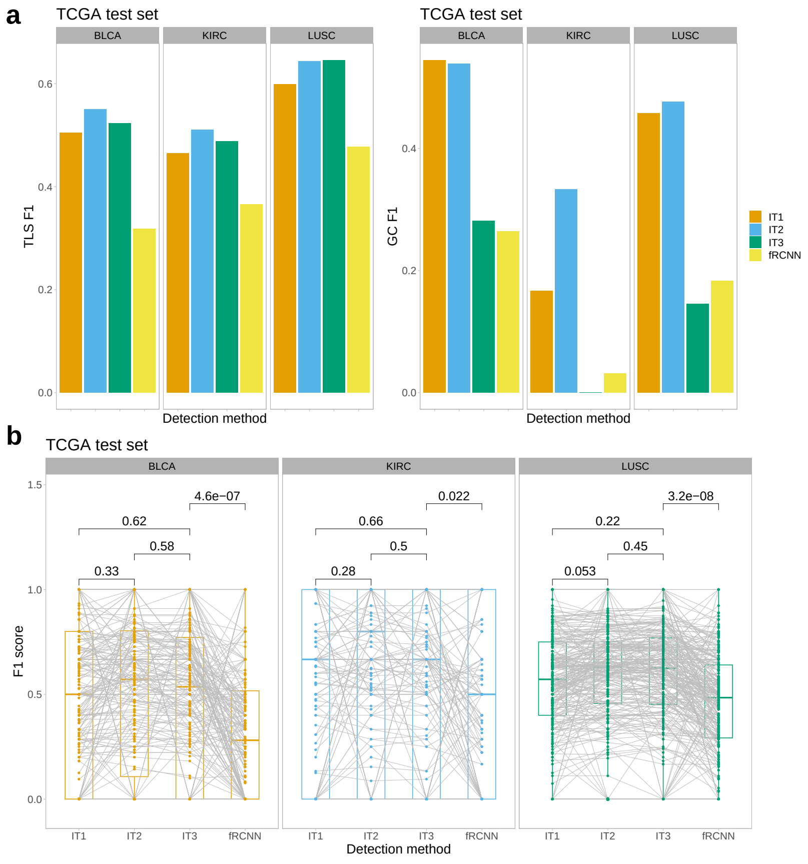


**Supplementary Figure 6** Hard-negative iteration comparisons. a: Comparison of overall F1-scores over three training iterations and Faster-RCNN for tertiary lymphoid structure (TLS) and germinal center (GC) detection in the Cancer Genome Atlas (TCGA) test set (n=767). b: Comparison of the per-slide F1-scores over three training iterations and Faster-RCNN for TLS detection in the TCGA test set (n=767). P values were obtained by paired Mann-Whitney U test. No correction for multiple testing was applied. Boxes span across the 25th and 75th percentiles, and whiskers (Tukey style) extend from the hinge to the largest value no further than 1.5 * interquartile range.

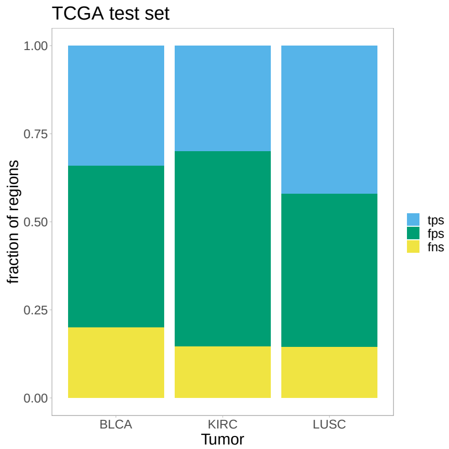


**Supplementary Figure 7** Proportions of false positive (fps), false negative (fns) and true positive (tps) regions detected by the HookNet-TLS model in each tumor type.

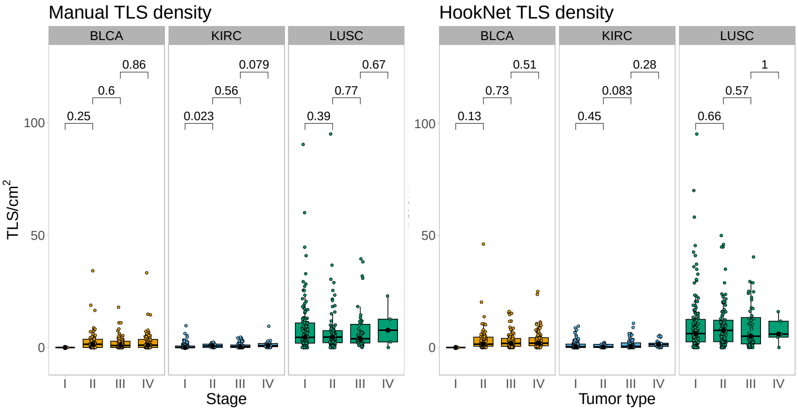


**Supplementary Figure 8** Comparison of tertiary lymphoid structure (TLS) densities across tumor stages in the Cancer Genome Atlas (TCGA) test set images (n-767) in different tumor types by Mann Whitney U test. No correction for multiple testing was applied. Boxes span across the 25th and 75th percentiles, and whiskers (Tukey style) extend from the hinge to the largest value no further than 1.5 * interquartile range.
