# Supplementary material for: Multi-resolution deep learning characterizes tertiary lymphoid structures and their prognostic relevance in solid tumors
Source: Commun Med (Lond). 2024 Jan 5;4:5. doi: 10.1038/s43856-023-00421-7 (PMC10770129; doi:10.1038/s43856-023-00421-7)
Supplement: Supplementary file 4 — Reporting Summary [file 43856_2023_421_MOESM4_ESM.pdf]

nature portfolio

Corresponding author(s): Mart van Rijthoven

Last updated by author(s): Nov 14, 2023

Reporting Summary

Nature Portfolio wishes to improve the reproducibility of the work that we publish. This form provides structure for consistency and transparency in reporting. For further information on Nature Portfolio policies, see our Editorial Policies and the Editorial Policy Checklist.

Statistics

For all statistical analyses, confirm that the following items are present in the figure legend, table legend, main text, or Methods section.

n/a	Confirmed	
☐	☒	The exact sample size (*n*) for each experimental group/condition, given as a discrete number and unit of measurement
☐	☒	A statement on whether measurements were taken from distinct samples or whether the same sample was measured repeatedly
☐	☒	The statistical test(s) used AND whether they are one- or two-sided<br>*Only common tests should be described solely by name; describe more complex techniques in the Methods section.*
☐	☒	A description of all covariates tested
☒	☐	A description of any assumptions or corrections, such as tests of normality and adjustment for multiple comparisons
☐	☒	A full description of the statistical parameters including central tendency (e.g. means) or other basic estimates (e.g. regression coefficient) AND variation (e.g. standard deviation) or associated estimates of uncertainty (e.g. confidence intervals)
☐	☒	For null hypothesis testing, the test statistic (e.g. *F*, *t*, *r*) with confidence intervals, effect sizes, degrees of freedom and *P* value noted<br>*Give P values as exact values whenever suitable.*
☒	☐	For Bayesian analysis, information on the choice of priors and Markov chain Monte Carlo settings
☒	☐	For hierarchical and complex designs, identification of the appropriate level for tests and full reporting of outcomes
☒	☐	Estimates of effect sizes (e.g. Cohen's *d*, Pearson's *r*), indicating how they were calculated

Our web collection on statistics for biologists contains articles on many of the points above.

Software and code

Policy information about availability of computer code

Data collection	QuPath v0.3.2<br>ASAP v2.1<br>WholeSlideData v0.0.16
Data analysis	QuPath v0.3.2<br>ASAP v2.1<br>WholeSlideData v0.0.16

For manuscripts utilizing custom algorithms or software that are central to the research but not yet described in published literature, software must be made available to editors and reviewers. We strongly encourage code deposition in a community repository (e.g. GitHub). See the Nature Portfolio guidelines for submitting code & software for further information.

Data

Policy information about availability of data

All manuscripts must include a data availability statement. This statement should provide the following information, where applicable:

- Accession codes, unique identifiers, or web links for publicly available datasets
- A description of any restrictions on data availability
- For clinical datasets or third party data, please ensure that the statement adheres to our policy

The TCGA dataset used in this study is publicly available and can be accessed through the TCGA repository (https://www.cancer.gov/ccg/research/genome-sequencing/tcga) In addition to the TCGA dataset we also created annotations that were specifically tailored to our study. These annotations and information on sample inclusion criteria and study-specific details, can be obtained upon request. Please contact: mart.vanrijthoven@gmail.com to request access to these annotations.

Human research participants

Policy information about studies involving human research participants and Sex and Gender in Research.

Reporting on sex and gender	We used data from the publicly available TCGA archive, there data on sex and gender is available. In our study we initially used this information as a covariate and found that it did not significantly correlate with our end points.
Population characteristics	We used data from the publicly available TCGA archive. Characteristics is based on the population included in TCGA archives for lung kidney and bladder cancer.
Recruitment	Data based on existing TCGA archives (TCGA-LUSC, TCGA-KIRC, TCGA-BLCA). See patient recruitment criteria on GDC data portal.
Ethics oversight	See TCGA data ethics oversight

Note that full information on the approval of the study protocol must also be provided in the manuscript.

Field-specific reporting

Please select the one below that is the best fit for your research. If you are not sure, read the appropriate sections before making your selection.

☒ Life sciences ☐ Behavioural & social sciences ☐ Ecological, evolutionary & environmental sciences

For a reference copy of the document with all sections, see nature.com/documents/nr-reporting-summary-flat.pdf

Life sciences study design

All studies must disclose on these points even when the disclosure is negative.

Sample size	See result section
Data exclusions	See result section
Replication	We made the code publicly available at: https://github.com/DIAGNijmegen/pathology-hooknet-tls
Randomization	n/a
Blinding	n/a

Reporting for specific materials, systems and methods

We require information from authors about some types of materials, experimental systems and methods used in many studies. Here, indicate whether each material, system or method listed is relevant to your study. If you are not sure if a list item applies to your research, read the appropriate section before selecting a response.

Materials & experimental systems

n/a	Involved in the study
☐	☒ Antibodies
☒	☐ Eukaryotic cell lines
☒	☐ Palaeontology and archaeology
☒	☐ Animals and other organisms
☐	☒ Clinical data
☒	☐ Dual use research of concern

Methods

n/a	Involved in the study
☒	☐ ChIP-seq
☒	☐ Flow cytometry
☒	☐ MRI-based neuroimaging

Antibodies

Antibodies used	See method section
Validation	See method section

Clinical data

Policy information about clinical studies

All manuscripts should comply with the ICMJE guidelines for publication of clinical research and a completed CONSORT checklist must be included with all submissions.

Clinical trial registration	The used data is not part of a clinical trial
Study protocol	As we used TCGA data, we relied on approval of TCGA study protocol
Data collection	See method section
Outcomes	The endpoint assesses the prognostic value of TLS assessed by AI. We compared this by human experts and showed comparable prognostic values in most cohorts.
